# Association between *IL‐1R2* polymorphisms and lung cancer risk in the Chinese Han population: A case–control study

**DOI:** 10.1002/mgg3.644

**Published:** 2019-03-20

**Authors:** Chaoying Wang, Chengsheng Zhang, Junnv Xu, Yongfu Li, Jie Wang, Hui Liu, Yueli Liu, Zhong Chen, Haifeng Lin

**Affiliations:** ^1^ Department of Medical Oncology The Second Affiliated Hospital of Hainan Medical University Haikou China; ^2^ Department of Nursing The Second Affiliated Hospital of Hainan Medical University Haikou China; ^3^ Department of Anatomy Hainan Medical University Haikou China; ^4^ Department of Pharmacology Hainan Medical University Haikou China; ^5^ Hainan Provincial Third People's Hospital Sanya China

**Keywords:** Case–control study, *IL‐1R2* gene, Lung cancer, Single‐nucleotide polymorphism

## Abstract

**Background:**

Interleukin‐1 receptor 2 (IL‐1R2), as an anti‐inflammatory cytokine, is involved in the pathogenesis and progression of lung cancer. However, the role of *IL‐1R2* polymorphisms in patients with lung cancer has yet to be fully elucidated.

**Methods:**

Six single‐nucleotide polymorphisms (SNPs) in *IL‐1R2* were genotyped in 259 patients and 346 healthy controls. We used the chi‐squared test, genetic model analysis, Haploview analysis, and multifactor dimensionality reduction (MDR) to evaluate the potential association between *IL‐1R2* polymorphisms and lung cancer susceptibility. Bioinformatics analyses were conducted to analyze the expression level of *IL‐1R2 *and its association with the overall survival of lung cancer.

**Results:**

Our results found that rs3218977‐GG was associated with a decreased risk of lung cancer (odds ratio [OR] = 0.39; 95% confidence interval [CI]: 0.17–0.87; *p* = 0.023), and rs2072472 had a significant risk‐increasing effect in the dominant model (AG + GG vs. AA: OR = 1.54; 95% CI: 1.09–2.20; *p* = 0.015). The MDR model also revealed that rs2072472 is the most influential risk factor of lung cancer (testing accuracy = 0.543; cross‐validation consistency = 10/10; *p* = 0.032). In addition, our results indicated that the *IL‐1R2 *mRNA level was downregulated in lung cancer patients, whereas the high expression of *IL‐1R2 *was related to a poor prognosis in lung cancer.

**Conclusions:**

Our results suggest that genetic variants of *IL‐1R2* may play a role in lung cancer susceptibility. Further population and functional validations of our findings are warranted.

## 1 INTRODUCTION

Lung cancer is the most frequently diagnosed cancer and is also the leading cause of cancer death worldwide (Torre et al., 2015). In China, an estimated 733,300 new lung cancer cases and 610,200 lung cancer deaths were diagnosed in 2015 with a rapidly increasing trend (Chen et al., 2016). According to a retrospective investigation, most lung cancer patients were diagnosed in the advanced stage, and the 5‐year survival rate for patients was only approximately 15.6% (Wood et al., 2012). It is known that lung cancer is a disease with unknown etiology which involves multiple environmental and genetic factors. Etiologically, tobacco smoking is the primary cause of lung cancer; other environmental factors, such as asbestos, heavy metal and air pollution, are also known as important risk factors (Dubey, Gupta, & Jain, 2016; Malhotra, Malvezzi, Negri, La Vecchia, & Boffetta, 2016). Additionally, individual variation, including age, sex, ethnicity, body weight, and especially single‐nucleotide polymorphisms (SNPs) associated with genetic susceptibility, exerts an important role in the etiology of lung cancer (T. Wang et al., 2014; Zhang et al., 2015; Zhou et al., 2015).

Chronic inflammation plays an important role in the development and progression of cancer, including proliferation, survival, and metastasis (Navarro et al., 2016; Rivasfuentes et al., 2015). Pro‐inflammatory cytokines (IL‐6, IL‐8, TNF‐α) and anti‐inflammatory cytokines (IL‐1RA, IL‐1R2) have been shown to be prospectively associated with increased lung cancer risk (Mario, Giovanny, Pedro, Norma, & Oscar, 2016). Interleukin‐1 (IL‐1) is a family of cytokines involved in inflammatory, immunological responses, and cancer formation by activating the expression of immune‐related genes (Sims & Smith, 2010). IL‐1R2, like IL‐1RA, acts as a natural inhibitor by competing with IL‐1R1 for IL‐1α and IL‐1β ligands to prevent the signal transduction of IL‐1 (Peters, Joesting, & Freund, 2013). *IL‐1R2* has been reported to regulate cell metabolism and respond to immune inflammation induced by many cytokines (Boraschi & Tagliabue, 2013). Notably, several studies have demonstrated *IL‐1R2 *abnormal expression in various cancers, such as lung cancer (Fennell et al., 2014), prostate cancer (Jones et al., 2013), and adrenocortical cancer (Szabo et al., 2014). These studies have suggested that *IL‐1R2* might be involved in the tumorigenesis and progression of cancers. Epidemiological studies have confirmed that *IL‐1R2* polymorphisms were associated with cancer susceptibility (Jones et al., 2013; Oelmann, Stein, Berdel, & Herbst, 2015). However, no previous study has investigated the association between lung cancer risk and *IL‐1R2* polymorphisms.

In the current study, we aimed to investigate *IL‐1R2* variants and their association with the susceptibility of lung cancer in a Chinese Han population using a case–control study.

## 2 MATERIALS AND METHODS

### 2.1 Study participants

We conducted a case–control study at the First Affiliated Hospital of Medical College of Xi′an Jiaotong University. We recruited 259 lung cancer patients and 346 healthy controls, and all subjects were genetically unrelated ethnic Han Chinese. Eligible cases were identified as lung cancer patients who were recently diagnosed by two pathologists according to the International Classification of Oncology, and the patients were enrolled with no limitations on tumor histology, grade of differentiation, or tumor stage. The patients were ascertained to have no history of cancer, infection, inflammation, or other autoimmune diseases. None of the patients had received radiotherapy or chemotherapy therapy before blood collection. Healthy controls were randomly recruited from the physical examination center of the First Affiliated Hospital of Medical College of Xi′an Jiaotong University during the same time that they had visited for an annual health examination. The exclusion criteria for the control group included any lung cancer family history of more than three generations, and chronic respiratory disease, tuberculosis, autoimmune disorders, and respiratory disorders.

### 2.2 Data collection

The demographic and clinical characteristics were obtained from a questionnaire and the medical records. Peripheral blood samples (about 5 ml) from all participants were collected and stored at −20°C for further laboratory analysis. This study protocol was approved by the Research Ethics Committee of First Affiliated Hospital of Xi′an Jiaotong University, and all experiments were conducted in accordance with the World Medical Association Declaration of Helsinki. Written informed consent was obtained from all subjects prior to the study's commencement.

### 2.3 SNPs selection and genotyping

The candidate SNPs in *IL‐1R2* were selected, which were based on the relevant studies of the SNPs associated with cancer or other diseases and combined with the minor allele frequency (MAF > 0.05) of the Han Chinese in the Beijing (CHB) population data from the 1,000 Genomes Project. Thus, six SNPs (rs11674595, rs4851527, rs719250, rs3218896, rs3218977, and rs2072472) were selected for further analysis. Genomic DNA extraction was performed from peripheral blood samples using the TIANamp Blood DNA Kit (Tiangen Biotech Ltd. Beijing, China). The DNA concentrations and purity were quantified using a NanoDrop 2000 system (Thermo Scientific, Waltham, MA, USA). The DNA samples were stored at −80°C until analysis. Genotyping of *IL‐1R2* polymorphisms was conducted by the Agena MassARRAY RS1000 system (San Diego, CA, USA) according to previously published protocols (Gabriel, Ziaugra, & Tabbaa, 2009). Primers for the amplification process and single‐base extension reactions were designed using Agena MassARRAY Assay Design 3.0 software. In addition, the data were analyzed using Agena Typer 4.0 Software. Genotyping was performed in a double‐blind test. For quality control, approximately 15% of the samples were randomly selected to carry out the repeated assays by different persons, and the results were 99.8% concordant.

### 2.4 Bioinformatics analysis

HaploReg v4.1 (https://pubs.broadinstitute.org/mammals/haploreg/haploreg.php) was used to predict the potential functions of the significant SNPs. *IL‐1R2* mRNA expression analysis was performed using GEPIA (http://gepia.cancer-pku.cn/) datasets. The prognostic significance of the mRNA expression of *IL‐1R2* gene in lung cancer was evaluated using the Kaplan–Meier plotter (http://kmplot.com/analysis/).

### 2.5 Data analysis

Statistical analyses were calculated using SPSS version 21.0 statistical software (SPSS, Chicago, IL, USA). Differences in the age distribution between the cases and controls were analyzed by the independent sample Student′s *t*‐test. Hardy–Weinberg equilibrium (HWE) analysis was carried out by Pearson′s goodness‐of‐fit chi‐squared test for the genotype frequency distribution of these SNPs in controls. The chi‐squared test/Fisher′s exact test was used to compare the distribution difference of allele and genotype frequencies in lung cancer patients and control subjects. The odds ratios (ORs), 95% confidence intervals (CIs), and *p*‐values were calculated using the logistic regression model adjustment for age and gender to assess the risk for lung cancer conferred by a certain allele and genotype. Furthermore, multiple inheritance models (codominant, dominant, recessive, and additive) were performed to estimate ORs using SNPstats software (http://bioinfo.iconcologia.net/snpstats/start.htm). Haploview software package (version 4.2) and SHEsis software platform were used to analyze the pairwise linkage disequilibrium, haplotype construction, and genetic association of polymorphism loci. Besides, multifactor dimensionality reduction (MDR version 3.0.2) was employed to evaluate the SNP–SNP interactions toward the risk of lung cancer (Leem & Park, 2017). The model was evaluated by cross‐validation consistency (CVC), testing balanced accuracy (denoted as testing bal. acc.), sign test, and statistical significance. All *p*‐values of statistical tests were two‐sided, and *p <*0.05 was deemed to indicate statistically significant differences.

## 3 RESULTS

### 3.1 Study participants

The study comprised of 259 lung cancer patients (199 males and 60 females) and 346 healthy controls (266 males and 80 females, Table 1). The mean age of the cases was 58.58 ± 9.74 years and the mean age of the controls was 50.84 ± 12.38 years. However, the result revealed that the age distribution was statistically significant differences (*p* < 0.001), suggesting that age may have an effect on the etiology of lung cancer. Lung cancer patients were consisted of 86 adenocarcinomas, 126 squamous cell carcinomas, and 47 small cell adenocarcinomas.

**Table 1:** Characteristics of patients with lung cancer and controls

Characteristics	Patients (*n* = 259)	Controls (*n* = 346)
Age, years	58.58 ± 9.74	50.84 ± 12.38
Gender		
Male	199	266
Female	60	80
Histology		
Adenocarcinoma	86	
Squamous cell	126	
Small cell carcinoma	47	
Stage		
I–II	67	
III–IV	192	
Lymphatic metastasis		
Yes	175	
No	84	

### 3.2 Potential regulatory role of selected SNPs

We used HaploRegv4.1 to annotate the potential function of these selected SNPs (Table 2). The results found that six intronic SNPs were associated with the regulation of promoter and/or enhancer histones, changed motifs, and selected eQTL hits, suggesting they might exert biological functions in this way in patients.

**Table 2:** Basic Information and the allele model analysis about* IL1R2* candidate SNPs

SNP ID	Location: position	Alleles (minor/major)	Frequency (MAF)		*p‐*value for HWE	OR (95% CI)	*p‐*value	HaploReg
			Cases	Controls				
rs11674595	Chr2:102610992	C/T	0.232	0.201	0.74	1.20 (0.91–1.58)	0.196	Promoter and Enhancer histone, Motifs changed, Selected eQTL hits
rs4851527	Chr2:102622376	A/G	0.290	0.312	0.71	0.90 (0.70–1.16)	0.409	Enhancer histone, Motifs changed, Selected eQTL hits
rs719250	Chr2:102623718	T/C	0.317	0.309	0.45	1.04 (0.81–1.33)	0.769	Promoter and Enhancer histone, Motifs changed, Selected eQTL hits
rs3218896	Chr2:102631652	C/T	0.145	0.155	1.00	0.92 (0.67–1.27)	0.621	Motifs changed, Selected eQTL hits
rs3218977	Chr2:102641201	G/A	0.219	0.247	0.08	0.85 (0.65–1.12)	0.252	Enhancer histone, Motifs changed, Selected eQTL hits
rs2072472	Chr2:102643019	G/A	0.247	0.200	0.18	**1.31 (1.00–1.73)**	0.051	Motifs changed, Selected eQTL hits

Bold indicates statistical significance.

SNP, single‐nucleotide polymorphism; MAF, minor allele frequency; OR, odds ratio; 95% CI, 95% confidence interval. *p*‐values were calculated using Pearson′s chi‐squared tests adjusted by gender and age.

* *p < *0.05 indicates statistical significance.

### 3.3 Hardy–Weinberg equilibrium and allelic frequency analyses

Six SNPs were successfully genotyped, and the call rate was above 99.17%. All SNPs among the control subjects were in accordance with HWE (*p* > 0.05, Table 2). There was no significant association of these SNPs in the *IL‐1R2* gene with lung cancer risk under the allelic model (Table 2).

### 3.4 Genetic model analysis of the association between *IL‐1R2* and lung cancer risk

The results of genetic models exhibited that rs3218977 and rs2072472 were associated with the risk of lung cancer. Rs3218977 moderately reduced lung cancer risk in the codominant (OR = 0.39; 95% CI: 0.17–0.87; *p* = 0.023) and recessive models (OR = 0.37; 95% CI: 0.17–0.81; *p* = 0.010). rs2072472 showed a risk‐increasing effect in the codominant (OR = 1.58; 95% CI: 1.09–2.29; *p* = 0.49), dominant (OR = 1.54; 95% CI: 1.09–2.20; *p* = 0.015), and log‐additive models (OR = 1.36; 95% CI: 1.02–1.81; *p* = 0.035). No significant associations were found between lung cancer risk and the remaining four SNPs (rs11674595, rs4851527, rs719250, and rs3218896; Table 3).

**Table 3:** Multiple inheritance model analysis of the association between the *IL1‐R2* SNPs and lung cancer risk

SNP ID	Model	Genotype	Control	Case	Crude analysis		Adjusted by age and gender	
					OR (95% CI)	*p*‐value	OR (95% CI)	*p*‐value
rs11674595	Codominant	T/T	221 (64.2%)	149 (58.0%)	1.00	0.260	1.00	0.300
		T/C	108 (31.4%)	97 (37.7%)	1.33 (0.94–1.88)		1.33 (0.93–1.92)	
		C/C	15 (4.4%)	11 (4.3%)	1.09 (0.49–2.43)		1.10 (0.47–2.59)	
	Dominant	T/T	221 (64.2%)	149 (58.0%)	1.00	0.120	1.00	0.140
		T/C–C/C	123 (35.8%)	108 (42.0%)	1.30 (0.93–1.81)		1.30 (0.92–1.85)	
	Recessive	T/T–T/C	329 (95.6%)	246 (95.7%)	1.00	0.960	1.00	0.980
		C/C	15 (4.4%)	11 (4.3%)	0.98 (0.44–2.17)		0.99 (0.42–2.31)	
	Log‐additive	—	—	—	1.20 (0.91–1.59)	0.190	1.21 (0.90–1.63)	0.210
rs4851527	Codominant	G/G	165 (47.8%)	130 (50.2%)	1.00	0.660	1.00	0.730
		G/A	145 (42.0%)	108 (41.7%)	0.95 (0.67–1.33)		0.94 (0.66–1.35)	
		A/A	35 (10.1%)	21 (8.1%)	0.76 (0.42–1.37)		0.78 (0.42–1.46)	
	Dominant	G/G	165 (47.8%)	130 (50.2%)	1.00	0.560	1.00	0.600
		G/A–A/A	180 (52.2%)	129 (49.8%)	0.91 (0.66–1.26)		0.91 (0.65–1.29)	
	Recessive	G/G–G/A	310 (89.9%)	238 (91.9%)	1.00	0.390	1.00	0.470
		A/A	35 (10.1%)	21 (8.1%)	0.78 (0.44–1.38)		0.80 (0.44–1.46)	
	Log‐additive	—	—	—	0.90 (0.70–1.16)	0.410	0.91 (0.70–1.18)	0.470
rs719250	Codominant	C/C	168 (48.7%)	125 (48.3%)	1.00	0.900	1.00	0.980
		T/C	141 (40.9%)	104 (40.1%)	0.99 (0.70–1.40)		0.98 (0.68–1.42)	
		T/T	36 (10.4%)	30 (11.6%)	1.12 (0.65–1.92)		1.04 (0.59–1.83)	
	Dominant	C/C	168 (48.7%)	125 (48.3%)	1.00	0.920	1.00	0.980
		T/C–T/T	177 (51.3%)	134 (51.7%)	1.02 (0.74–1.40)		1.00 (0.71–1.40)	
	Recessive	C/C–T/C	309 (89.6%)	229 (88.4%)	1.00	0.660	1.00	0.870
		T/T	36 (10.4%)	30 (11.6%)	1.12 (0.67–1.88)		1.04 (0.61–1.79)	
	Log‐additive	—	—	—	1.04 (0.82–1.32)	0.780	1.01 (0.78–1.30)	0.950
rs3218896	Codominant	T/T	246 (71.3%)	191 (73.8%)	1.00	0.710	1.00	0.820
		T/C	91 (26.4%)	61 (23.6%)	0.86 (0.59–1.26)		0.88 (0.59–1.31)	
		C/C	8 (2.3%)	7 (2.7%)	1.13 (0.40–3.16)		0.99 (0.34–2.87)	
	Dominant	T/T	246 (71.3%)	191 (73.8%)	1.00	0.510	1.00	0.550
		T/C–C/C	99 (28.7%)	68 (26.2%)	0.88 (0.62–1.27)		0.89 (0.61–1.31)	
	Recessive	T/T–T/C	337 (97.7%)	252 (97.3%)	1.00	0.760	1.00	0.960
		C/C	8 (2.3%)	7 (2.7%)	1.17 (0.42–3.27)		1.03 (0.36–2.95)	
	Log‐additive	—	—	—	0.92 (0.67–1.27)	0.620	0.92 (0.66–1.28)	0.620
rs3218977	Codominant	A/A	201 (58.4%)	154 (60.2%)	1.00	0.120	1.00	0.023*
		G/A	116 (33.7%)	92 (35.9%)	1.04 (0.73–1.46)		1.19 (0.82–1.71)	
		G/G	27 (7.8%)	10 (3.9%)	0.48 (0.23–1.03)		**0.39 (0.17–0.87)**	
	Dominant	A/A	201 (58.4%)	154 (60.2%)	1.00	0.670	1.00	0.950
		G/A–G/G	143 (41.6%)	102 (39.8%)	0.93 (0.67–1.29)		1.01 (0.71–1.44)	
	Recessive	A/A–G/A	317 (92.2%)	246 (96.1%)	1.00	0.042*	1.00	0.010*
		G/G	27 (7.8%)	10 (3.9%)	0.48 (0.23–1.00)		**0.37 (0.17–0.81)**	
	Log‐additive	—	—	—	0.86 (0.66–1.12)	0.260	0.87 (0.66–1.16)	0.350
rs2072472	Codominant	A/A	225 (65.2%)	147 (56.8%)	1.00	0.110	1.00	0.049*
		A/G	102 (29.6%)	96 (37.1%)	**1.44 (1.02–2.04)**		**1.58 (1.09–2.29)**	
		G/G	18 (5.2%)	16 (6.2%)	1.36 (0.67–2.75)		1.36 (0.64–2.87)	
	Dominant	A/A	225 (65.2%)	147 (56.8%)	1.00	0.035*	1.00	0.015*
		A/G–G/G	120 (34.8%)	112 (43.2%)	**1.43 (1.03–1.99)**		**1.54 (1.09–2.20)**	
	Recessive	A/A–A/G	327 (94.8%)	243 (93.8%)	1.00	0.610	1.00	0.690
		G/G	18 (5.2%)	16 (6.2%)	1.20 (0.60–2.39)		1.16 (0.56–2.42)	
	Log‐additive	—	—	—	1.30 (0.99–1.70)	0.056	**1.36 (1.02–1.81)**	0.035*

Bold indicates statistical significance. *p*‐values were calculated using Pearson′s chi‐squared tests adjusted by gender and age. SNP, single‐nucleotide polymorphism; OR, odds ratio; 95% CI, 95% confidence interval

* *p < *0.05 indicates statistical significance.

### 3.5 Stratification analysis

Lung cancer cases were further stratified into histological subgroups (Table 4). We found a significant association between rs719250 polymorphism and lung small cell carcinoma (allele, OR = 1.97, 95% CI: 1.27–3.05, *p* = 0.002; codominant, OR = 2.64, 95% CI: 1.29–5.50, *p* = 0.007; dominant, OR = 2.84, 95% CI: 1.40–5.73, *p* = 0.002; additive model, OR = 1.97, 95% CI: 1.26–3.09, *p* = 0.003). Moreover, individuals carrying rs3218977‐GG genotype showed a significant protective effect for lung squamous cell carcinoma (GG vs. AA, OR = 0.07, 95% CI: 0.01–0.58, *p* = 0.002; and GG vs. AA‐GA, OR = 0.07, 95% CI: 0.01–0.54, *p* < 0.001).

**Table 4:** Stratified analysis of *IL1R2* polymorphisms and lung cancer susceptibility

SNP ID	Model	Genotype	Control	Adenocarcinoma			Squamous cell carcinoma			Small cell carcinoma		
				Case	OR (95% CI)	*p*‐value	Case	OR (95% CI)	*p*‐value	Case	OR (95% CI)	*p*‐value
rs719250	Allele	C	477 (69.1%)	180 (71.4%)	1.00	0.497	116 (67.4%)	1.00	0.669	50 (53.2%)	1.00	0.002*
		T	213 (30.9%)	72 (28.6%)	0.90 (0.65–1.23)		56 (32.6%)	1.08 (0.76–1.55)		44 (46.8%)	**1.97 (1.27–3.05)**	
	Codominant	C/C	168 (48.7%)	64 (50.8%)	1.00	0.600	42 (48.8%)	1.00	0.630	12 (25.5%)	1.00	0.007*
		T/C	141 (40.9%)	52 (41.3%)	1.03 (0.66–1.61)		32 (37.2%)	0.84 (0.48–1.47)		26 (55.3%)	**2.64 (1.26–5.50)**	
		T/T	36 (10.4%)	10 (7.9%)	0.70 (0.32–1.51)		12 (13.9%)	1.24 (0.55–2.80)		9 (19.1%)	**3.60 (1.38–9.36)**	
	Dominant	C/C	168 (48.7%)	64 (50.8%)	1.00	0.820	42 (48.8%)	1.00	0.750	12 (25.5%)	1.00	0.002*
		T/C–T/T	177 (51.3%)	62 (49.2%)	0.95 (0.62–1.46)		44 (51.2%)	0.92 (0.55–1.55)		35 (74.5%)	**2.84 (1.40–5.73)**	
	Recessive	C/C–T/C	309 (89.6%)	116 (92.1%)	1.00	0.310	74 (86%)	1.00	0.470	38 (80.8%)	1.00	0.099
		T/T	36 (10.4%)	10 (7.9%)	0.69 (0.32–1.45)		12 (13.9%)	1.34 (0.62–2.92)		9 (19.1%)	2.07 (0.91–4.73)	
	Log‐additive	—	—	—	0.90 (0.66–1.25)	0.540	—	1.02 (0.70–1.50)	0.910	—	**1.97 (1.26–3.09)**	0.003*
rs3218977	Allele	A	518	183	1.00	0.515	137	1.00	0.146	76	1.00	0.122
		G	170	67	1.12 (0.80–1.55)		33	0.73 (0.48–1.11)		16	0.64 (0.36–1.13)	
	Codominant	A/A	201 (58.4%)	69 (55.2%)	1.00	0.590	53 (62.4%)	1.00	0.002*	32 (69.6%)	1.00	0.220
		G/A	116 (33.7%)	45 (36%)	1.27 (0.80–2.01)		31 (36.5%)	1.21 (0.69–2.11)		12 (26.1%)	0.71 (0.35–1.45)	
		G/G	27 (7.8%)	11 (8.8%)	1.05 (0.48–2.31)		1 (1.2%)	**0.07 (0.01–0.58)**		2 (4.3%)	0.33 (0.07–1.53)	
	Dominant	A/A	201 (58.4%)	69 (55.2%)	1.00	0.350	53 (62.4%)	1.00	0.650	32 (69.6%)	1.00	0.150
		G/A–G/G	143 (41.6%)	56 (44.8%)	1.22 (0.80–1.88)		32 (37.6%)	0.88 (0.52–1.51)		14 (30.4%)	0.62 (0.31–1.21)	
	Recessive	A/A–G/A	317 (92.2%)	114 (91.2%)	1.00	0.930	84 (98.8%)	1.00	<0.001*	44 (95.7%)	1.00	0.150
		G/G	27 (7.8%)	11 (8.8%)	0.96 (0.45–2.08)		1 (1.2%)	**0.07 (0.01–0.54)**		2 (4.3%)	0.36 (0.08–1.67)	
	Log‐additive	—	—	—	1.12 (0.81–1.54)	0.510	—	0.70 (0.45–1.09)	0.110	—	0.64 (0.37–1.10)	0.091

Note

Bold indicates statistical significance. *p*‐values were calculated using Pearson′s chi‐squared tests adjusted by gender and age. SNP, single‐nucleotide polymorphism; OR, odds ratio; 95% CI, 95% confidence interval.

* *p < *0.05 indicates statistical significance.

We segregated patients according to clinical stage (I–II vs. III–IV) and lymphatic metastasis (non‐metastasis vs. metastasis). We found that rs11674595 C allele was highly represented in patients with III–IV tumor stage as compared to patients with I–II tumor stage (allele, OR = 1.77, 95% CI: 1.05–2.99, *p* = 0.029; additive, OR = 1.76, 95% CI: 1.04–2.98, *p* = 0.027; Table 5). No significant association was observed between tumor metastasis and *IL‐1R2* variants.

**Table 5:** Relationship of clinical stage with* IL1‐R2* polymorphisms in lung cancer patients adjusted by gender and age

SNP ID	Model	Genotype	I–II	III–IV	OR (95%CI)	*p‐*value
rs11674595	Allele	T	111 (84.1%)	286 (74.9%)	1.00	0.029*
		C	21 (15.9%)	96 (25.1%)	**1.77 (1.05–2.99)**	
	Codominant	T/T	46 (69.7%)	108 (56.5%)	1.00	0.065
		T/C	19 (28.8%)	70 (36.6%)	1.56 (0.84–2.88)	
		C/C	1 (1.5%)	13 (6.8%)	5.55 (0.70–43.77)	
	Dominant	T/T	46 (69.7%)	108 (56.5%)	1.00	0.060
		T/C‐C/C	20 (30.3%)	83 (43.5%)	1.76 (0.97–3.20)	
	Recessive	T/T‐T/C	65 (98.5%)	178 (93.2%)	1.00	0.066
		C/C	1 (1.5%)	13 (6.8%)	4.76 (0.61–37.20)	
	Log‐additive	—	—	—	**1.76 (1.04–2.98)**	0.027*

Bold indicates statistical significance. *p*‐values were calculated with Pearson′s chi‐squared tests. OR, odds ratio; 95% CI, 95% confidence interval.

* *p ≤ *0.05 indicates statistical significance.

### 3.6 Haplotype analysis

**Figure 1:**
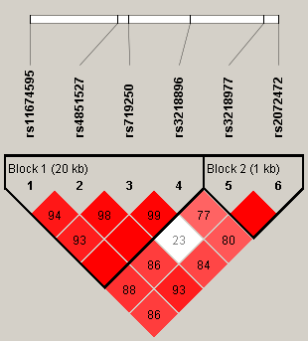
Haplotype block map for six SNPs in the *IL‐1R2* gene. The numbers inside the diamonds indicate the D′ for pairwise analyses

Additionally, haplotype analysis was conducted, and the result of linkage disequilibrium revealed the existence of two blocks in *IL‐1R2* SNPs (Figure 1). The haplotype frequencies of four SNP haplotypes (rs11674595, rs4851527, rs719250, and rs3218896) and two SNP haplotypes (rs3218977 and rs2072472) in the case and control groups were shown in Table S1. Haplotypes with < 1% frequency were excluded from haplotype analysis. Haplotype‐based logistic regression adjusted by age and gender was performed within the case–control cohort; however, the results revealed no significant association of the haplotypes with the risk of lung cancer (*p* > 0.05, Table S1).

### 3.7 MDR analysis

The results of MDR analysis were summarized in Table 6. rs2072472 was the most influential attributor for lung cancer risk in the single‐locus model (testing accuracy = 0.5428, CVC = 10/10, *p* = 0.0321), a finding that is consistent with the results of previous studies. Moreover, the three‐locus interaction model (rs719250, rs3218977, and rs2072472) was found to be the best in the multilocus model (testing accuracy = 0.5409, CVC = 10/10, *p* = 0.0002). The accumulated effect of all risk factors of SNPs (OR = 2.50; 95% CI: 1.79–3.47; *p* < 0.001) contributed to increased susceptibility of lung cancer compared with the single‐risk genotype.

**Table 6:** SNP–SNP interaction models of the *IL‐1R2* gene analyzed by the MDR method

Model	Training Bal. Acc.	Testing Bal. Acc.	CVC	OR (95% CI)	*p‐*value
rs2072472	0.543	0.543	10/10	1.43 (1.03–1.99)	0.0321*
rs11674595, rs2072472	0.559	0.492	6/10	1.58 (1.14–2.21)	0.0064*
rs719250, rs3218977, rs2072472	0.577	0.541	10/10	1.85 (1.33–2.56)	0.0002*
rs11674595, rs719250, rs3218977, rs2072472	0.593	0.527	10/10	2.09 (1.51–2.91)	<0.0001*
rs11674595, rs4851527, rs719250, rs3218977, rs2072472	0.605	0.510	6/10	2.32 (1.66–3.22)	<0.0001*
rs11674595, rs4851527, rs719250, rs3218896, rs3218977, rs2072472	0.615	0.506	10/10	2.49 (1.79–3.47)	<0.0001*

Bold indicates statistical significance.

*p*‐values were calculated using chi‐squared tests. MDR, multifactor dimensionality reduction; Bal. Acc., Balanced accuracy; CVC, Cross‐validation consistency; OR, odds ratio; 95% CI, 95% confidence interval.

* *p* < 0.05 indicates statistical significance.

### 3.8 Bioinformatics analysis of *IL‐1R2* expression and prognosis

*IL‐1R2* gene was downregulated in lung adenocarcinoma (LUAD, *p* < 0.01) and lung squamous cell carcinoma (LUSC, *p* < 0.01) based on GEPIA database (Figure S1). Moreover, *IL‐1R2* high expression was found to be associated with lung cancer patients (hazard ratio = 1.37; 95% CI: 1.21–1.55; *p* = 1.1e‐06) based on Kaplan–Meier plotter (Figure S2).

## 4 DISCUSSION

Many lines of evidence have suggested that genetic factors play an important role in determining the susceptibility to lung cancer (Galvan et al., 2010). In this case–control study, we successfully genotyped six SNPs in the* IL1‐R2* gene and found that rs2072472, rs719250, rs11674595, and rs3218977 were related to the risk of lung cancer. Furthermore, the results of the MDR analysis also revealed a significant association between the SNP–SNP interactions and lung cancer susceptibility. The findings further highlight that the polymorphisms in the *IL1‐R2* gene may contribute to lung cancer susceptibility in the Chinese Han population. To the best of our knowledge, this is the first study to explore the relationship between* IL1‐R2* polymorphisms and lung cancer risk in the Chinese Han population. Our study provides evidence about the potential role of the *IL1‐R2 *variants in lung cancer risk. Combined with the previous studies, this association may be a promising starting point for a functional profile of the *IL1‐R2 *gene and increased understanding of the biological processes associated with lung cancer formation and progression.

Chronic inflammation and cytokines are believed to contribute to tumor growth, progression, and even immunosuppression (Mantovani, Allavena, Sica, & Balkwill, 2008; de Visser, Eichten, & Coussens, 2006). The functions of interleukin‐1 involve the signal transduction pathways of GTP‐binding proteins and activation of MAP kinase, IκB/NFκB, and JNK family members (Martin & Wesche, 2002). IL‐1R2, as a member of the IL1 family, is located on the long arm of chromosome 2 at band 2q12 in humans. As a decoy receptor, IL‐1R2 is a natural inhibitor of IL1 and plays important roles in tumor‐associated inflammation and immune regulation. Upregulation of *IL‐1R2* has been observed in different cancers, such as pancreatic ductal adenocarcinoma (Rückert et al., 2010), prostate cancer (Ricote et al., 2010), and ovarian cancer (Laios et al., 2008), supporting the involvement of *IL‐1R2* in cancer progression. *IL‐1R2* was shown to be rapidly upregulated in human regulatory T cells (Tregs) and is correlated with a poor prognosis in lung adenocarcinoma (Frances, Lina, & Derya, 2010; Guo et al., 2018). In silico, we found that *IL‐1R2* gene expression is downregulated in lung cancer, whereas high expression of *IL‐1R2 *is associated with a poor overall survival for lung cancer. We speculated that *IL‐1R2* plays an important role in the progress and prognosis of lung cancer, but more studies are needed to validate.

Studies based on variants in the *IL‐1R2 *gene are infrequent, and there is no finding of the association between the *IL‐1R2* SNPs and lung cancer risk in previous studies. Given the expression and survival data of *IL‐1R2* in lung cancer, polymorphisms in the *IL‐1R2 *gene might contribute to lung cancer risk. Our results found that rs3218977 in *IL‐1R2* was associated with a decreased risk of lung cancer, especially in patients with lung squamous cell carcinoma. rs2072472 polymorphism increased 1.77‐fold risk of lung cancer, and rs719250 polymorphism increased 1.77‐fold risk of lung small cell carcinoma. Moreover, rs11674595 polymorphism was a significant risk factor for patients with III‐IV stage. Given that lung cancer represents a complex disorder, SNP–SNP interaction studies may help discover the risk factors for lung cancer. Accordingly, we performed the MDR to determine the potential SNP–SNP interactions between these SNPs in the *IL1R2* gene. The analysis of the SNP–SNP interactions showed a strong interaction between these SNPs regarding susceptibility to lung cancer. Several studies provided increasing evidence to support that intronic SNPs confer susceptibilities by affecting the binding of transcription factor binds and/or RNA splicing (Seo et al., 2013; D. Wang & Sadee, 2016; Zhao et al., 2012). However, the mechanisms on the biological function of these SNPs in* IL1R2 *are unknown and need more functional studies to explore.

Several limitations of this investigation should be acknowledged. Primarily, the potential function of *IL‐R2* polymorphisms and the expression data and survival data of *IL‐R2* were from the database. The expression analysis of *IL‐1R2* mRNA and annotation of the functional significance of the variants are needed to clarify the genetic mechanism underlying lung cancer in the future. In addition, some exposure information (such as smoking and drinking) was missing. More well‐designed population‐based studies should be conducted to further investigate the interactions with environmental factors, and subgroup analysis should be performed for histology.

## 5 CONCLUSION

In summary, this is an exploratory study concerning the association of *IL‐1R2* SNPs with the susceptibility of lung cancer. *IL‐1R2* rs2072472 polymorphism increased the risk of lung cancer; conversely, the rs3218977 polymorphism reduced the susceptibility of lung cancer. Future studies are necessary to investigate the detailed mechanisms of the associated variants that affect the expression and function of the *IL‐1R2* gene.

## CONFLICT OF INTEREST

The authors declare that they have no conflict of interest.
