# Supplementary figures and images for: Association between IL‐1R2 polymorphisms and lung cancer risk in the Chinese Han population: A case–control study
Source: Mol Genet Genomic Med. 2019 Mar 20;7(5):e644. doi: 10.1002/mgg3.644 (PMC6503014; doi:10.1002/mgg3.644)

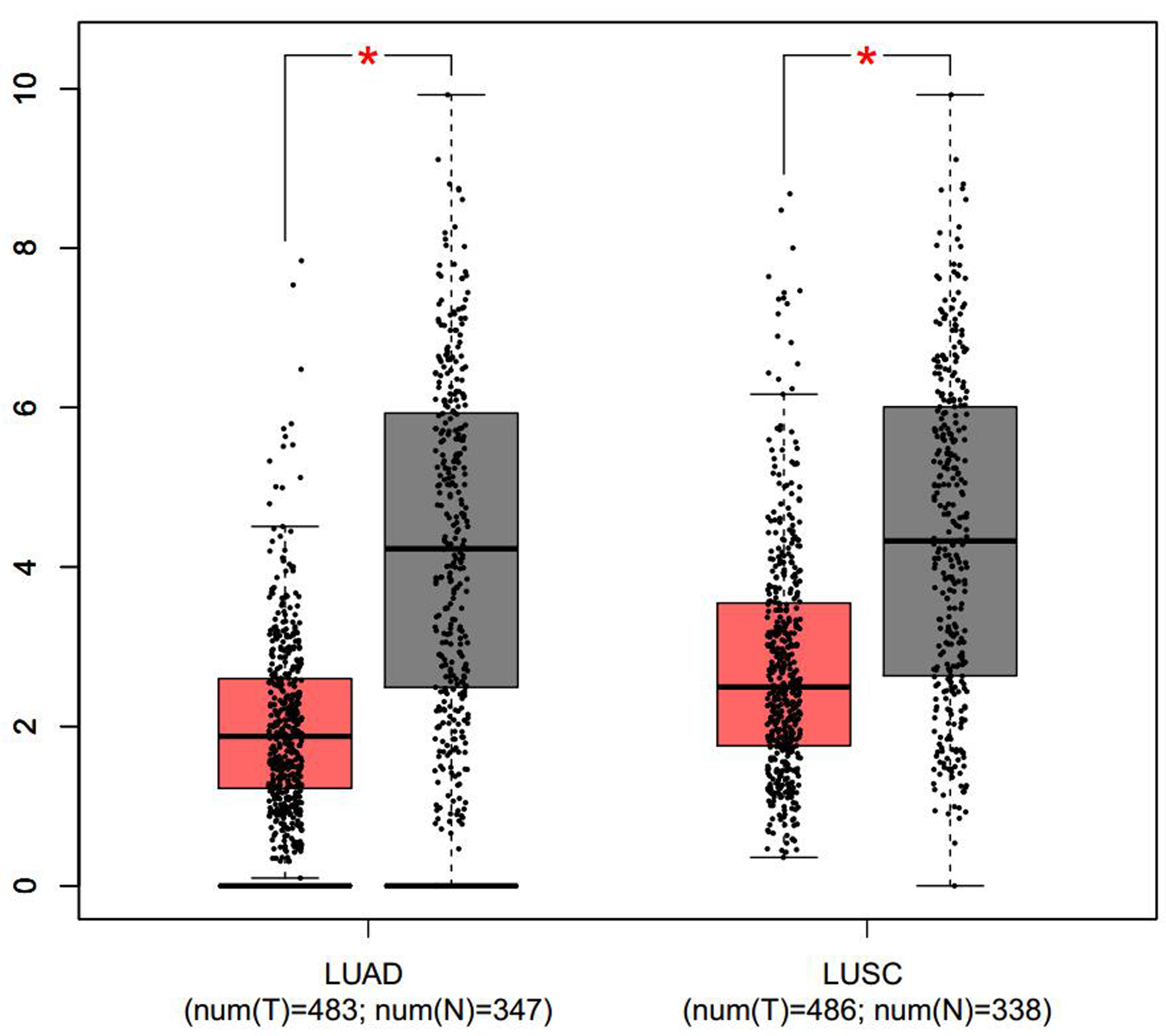

Supplement: Supplementary file 1 [file MGG3-7-e644-s001.tif]

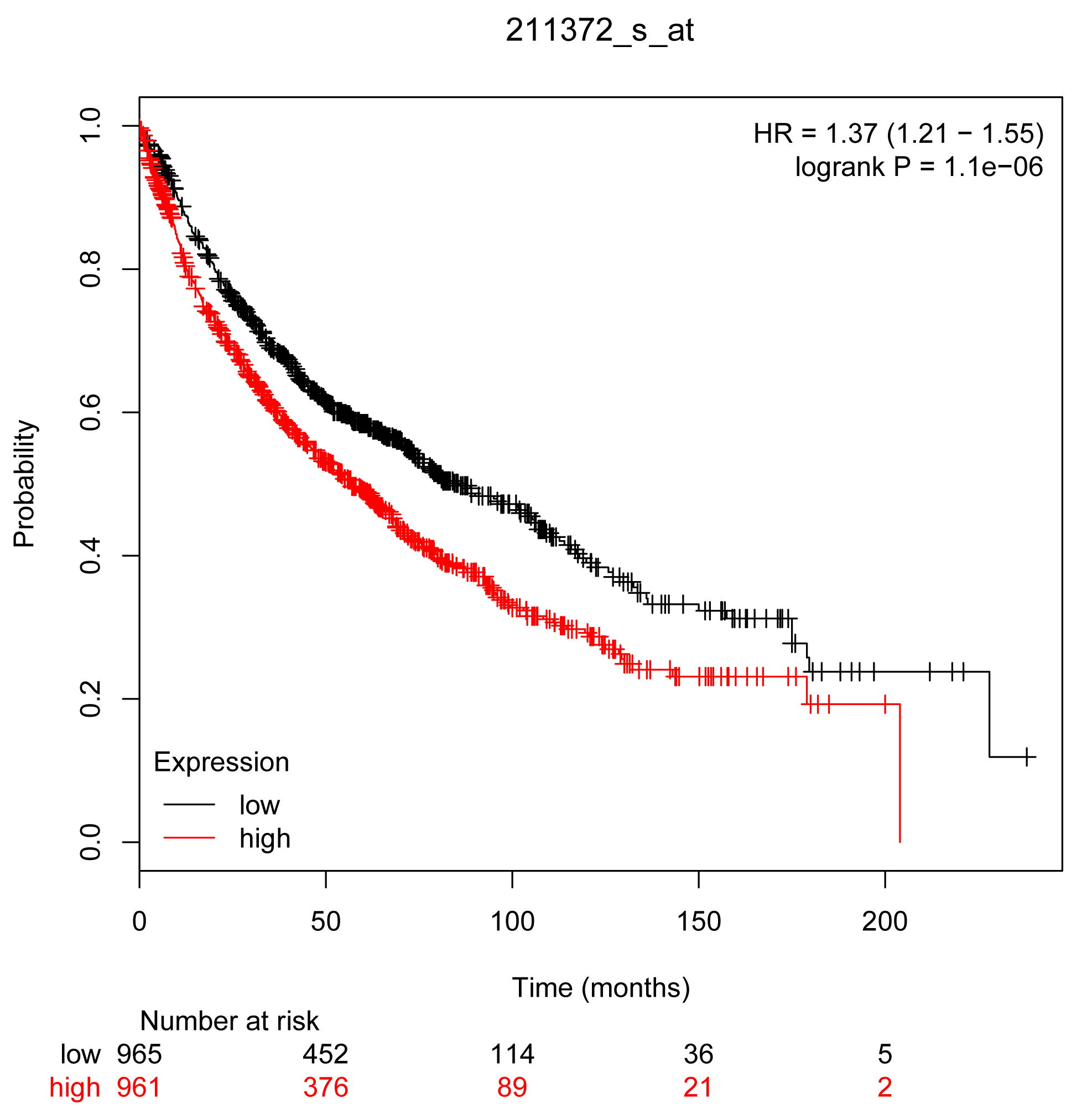

Supplement: Supplementary file 2 [file MGG3-7-e644-s002.tif]
