# Supplementary material for: Association between IL‐1R2 polymorphisms and lung cancer risk in the Chinese Han population: A case–control study
Source: Mol Genet Genomic Med. 2019 Mar 20;7(5):e644. doi: 10.1002/mgg3.644 (PMC6503014; doi:10.1002/mgg3.644)
Supplement: Supplementary file 3 [file MGG3-7-e644-s003.docx]

**Supplementary Table 1. Haplotype frequencies and their associations with lung cancer risk.**

|  | **Haplotype** | **Frequency** | | **χ^2^** | ***p* value** | **Crude analysis** | | **Adjusted by age and gender** | |
| --- | --- | --- | --- | --- | --- | --- | --- | --- | --- |
|  |  | **case** | **control** |  |  | **OR (95% CI)** | ***p*** | **OR (95% CI)** | ***p*** |
| **Block 1** | TACT | 0.286 | 0.307 | 0.603 | 0.44 | 1.00 |  | 1.00 |  |
|  | CGCT | 0.227 | 0.196 | 1.733 | 0.19 | 1.26 (0.91–1.75) | 0.170 | 1.25 (0.88–1.77) | 0.210 |
|  | TGCT | 0.167 | 0.186 | 0.663 | 0.42 | 0.96 (0.68–1.36) | 0.830 | 0.99 (0.69–1.43) | 0.960 |
|  | TGTT | 0.167 | 0.149 | 0.712 | 0.40 | 1.21 (0.85–1.73) | 0.300 | 1.16 (0.80–1.70) | 0.430 |
|  | TGTC | 0.145 | 0.154 | 0.200 | 0.66 | 1.01 (0.70–1.45) | 0.960 | 1.01 (0.69–1.47) | 0.980 |
|  | **Global haplotype association p value:** | | | | |  | 0.610 |  | 0.780 |
| **Block 2** | AA | 0.536 | 0.553 | 0.354 | 0.55 | 1.00 |  | 1.00 |  |
|  | GA | 0.217 | 0.247 | 1.471 | 0.23 | 0.91 (0.69–1.20) | 0.500 | 0.94 (0.70–1.27) | 0.690 |
|  | AG | 0.247 | 0.200 | 3.823 | 0.05 | 1.26 (0.95–1.67) | 0.100 | 1.34 (0.99–1.80) | 0.058 |
|  | **Global haplotype association p value:** | | | | |  | 0.130 |  | 0.099 |

Block 1 comprises the four closely linked SNPs rs11674595, rs4851527, rs719250 and rs3218896. Block 2 comprises the two closely linked SNPs rs3218977 and rs2072472. OR = odds ratio; 95% CI = 95% confidence interval. *p* values were calculated using Pearson′s χ^2^ tests adjusted by gender and age; *p* < 0.05 indicates statistical significance.
